# Role of *KCNQ1OT1* / *miR-27b-5p* in Modulating Dihydrolipoamide S-acetyltransferase (DLAT): Insights into Cuproptosis in Hepatocellular carcinoma

**DOI:** 10.1007/s12011-026-04999-6

**Published:** 2026-02-18

**Authors:** Ahmed S. Elkateb, Heba Taha, Tamer Abou Elela, Rehab Ahmed Abdel- Hamid, Sahar A. Ali, Hanaa B. Atya

**Affiliations:** 1 Biochemistry and Molecular Biology Department, Faculty of Pharmacy, Helwan University, P.O. Box 11795, Cairo, Egypt; 2 Department of Hepatology & Gastroenterology, National Hepatology and Tropical Medicine Research Institute (NHTMRI), Cairo, Egypt

**Keywords:** Cuproptosis, HCC, DLAT, KCNQ1OT, miR-27b-5p, Sorafenib, Regorafenib

## Abstract

Hepatocellular carcinoma (HCC) poses a significant global health challenge due to late diagnosis and limited treatment effectiveness. Cuproptosis, a newly identified form of copper-induced mitochondrial cell death, is emerging as a promising therapeutic target, with Dihydrolipoamide S-acetyltransferase (DLAT) playing a key role in protein aggregation during this process. This study investigates the long non-coding RNA *KCNQ1OT1/miR-27b-5p*/DLAT regulatory axis’ involvement in cuproptosis and HCC progression, emphasizing its potential for diagnostic and therapeutic monitoring. Accordingly, a total of 180 participants were divided into three main groups: A healthy control group, untreated HCC group (naïve, NV) and treated HCC group which further subclassified into HCC patients treated with Sorafenib (Sor) or treated with Regorafenib (Rog). The regulatory axis *KCNQ1OT1*/*miR-27b-5p*/DLAT was predicted through bioinformatics analysis. Expression levels of *KCNQ1OT1*, *miR-27b-5p,* and cuproptosis-related genes (*ATP7A, ATP7B, SLC31A1*) were assessed using RT-qPCR. Serum DLAT was measured through ELISA technique.

The NV group showed elevated serum DLAT levels, *KCNQ1OT1* expression, and ATP7A/B levels, while *miR-27b-5p*, SLC31A1 expression, and serum Glutathione (GSH) levels were reduced, indicating impaired cuproptosis. In contrast, the treated groups (Sor & Rog) demonstrated lower DLAT, *KCNQ1OT1*, and *ATP7A/B* levels, alongside higher *miR-27b-5p, SLC31A1*, and GSH levels, suggesting copper accumulation and activated cuproptosis. ROC and correlation analyses indicate that *KCNQ1OT1*, *miR-27b-5p,* and DLAT constitute a regulatory axis, potentially serving as diagnostic and therapeutic monitoring biomarkers in HCC.

**Graphical Abstract:**

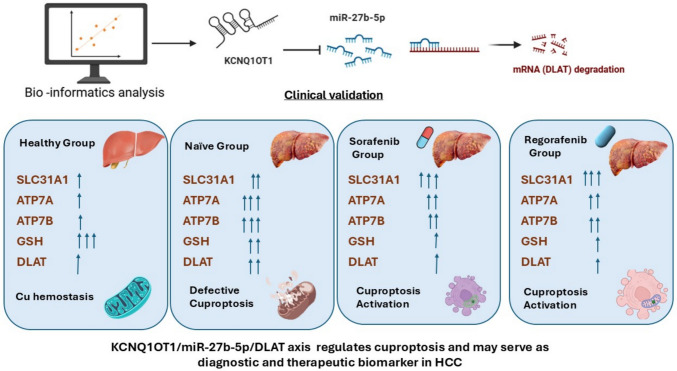

**Supplementary Information:**

The online version contains supplementary material available at 10.1007/s12011-026-04999-6.

## Introduction

In 2022, the World Health Organization indicated that hepatocellular carcinoma (HCC) was the third leading cause of cancer-related death globally, resulting in approximately 758,725 deaths [1]. Despite recent expansions in HCC diagnosis and treatment, 80% of patients are diagnosed at an advanced stage which is associated with low survival rate and few possible effective chemotherapeutic options [2].

One of the systemic drugs utilized in advanced HCC is sorafenib (Nexavar®), multi-kinase inhibitor [3]. Sorafenib reduces the growth of HCC by triggering various cell death processes, including apoptosis, ferroptosis, and autophagy. However, after six months, treatment resistance often appears, and only around 30% of patients initially respond well [4]. For those who have tolerated sorafenib but experienced resistance, regorafenib is often prescribed as a second-line systemic treatment [5].

The discovery of new cell death mechanisms like ferroptosis, necroptosis, and pyroptosis has strengthened our understanding of the carcinogenic mechanisms in HCC. This knowledge aids in diagnosis, elucidates the pathogenesis of HCC, and clarifies the mechanism of action of sorafenib and regorafenib [6].

In 2022, Tsvetkov, P et al. has reported noteworthy research on copper-mediated cell death, namely ‘cuproptosis’ [7]. Normally The body uses the copper in many physiological processes, like detoxification of oxygen free radicals, iron transport and mitochondrial respiration [8] In addition, the body maintains copper concentration within normal ranges via active homeostatic mechanism: ATPase Copper Transporting Alpha (*ATP7A*), ATPase Copper Transporting Beta (*ATP7B*) and Solute Carrier Family 31 Member 1 (*SLC31A1*). These transporters use the ATP to move copper across cell membranes to prevent free copper overload, which can damage cells [9]. Furthermore, changes in intracellular copper levels could contribute to the development of cancer [9].

In terms of mechanism, excess copper causes cuproptosis, and one of key proteins in this process is dihydrolipoamide S-acetyltransferase (DLAT). DLAT is a key component of the pyruvate dehydrogenase complex that is required for tricarboxylic acid cycle function. Elevated copper ions cause DLAT to form oligomerized DLAT proteins and lose its function, leading to protein proteotoxic stress and cell death [8]. Recently, many bioinformatics and invitro studies showed that DLAT can be a biomarker for prognosis in different cancers like gastric cancer, pancreatic cancer, and renal cell carcinoma [8, 10, 11]. Furthermore, Guan et al. showed that high DLAT's was correlated with poor survival in liver cancer [12]. While Wang et al. showed that sorafenib can intensify elesclomol drug action (copper ionophore) in liver cancer via stimulating lipoylated DLAT aggregation and inhibiting intracellular reduced glutathione (GSH) synthesis, promoting cuproptosis [13]. But until now, the regorafenib’s role in regulating cuproptosis remains unknown.

Noncoding RNAs (ncRNAs) are functional RNA that regulate the expression of the proteins involved in many cellular processes, cell death mechanisms and disease mechanisms like HCC [14]. MicroRNAs are a subtype of non-coding RNA (ncRNA) with a length of 21–24 nucleotides that controls post-transcriptional regulation of genes. They interact with specific mRNAs by complementary base-pairing, which affects the target mRNA molecule's stability or translation [15]. Long non-coding RNAs (lncRNAs), a different subtype of ncRNA, are transcripts longer than 200 nucleotides. These lncRNAs act as sponges by binding to and sequestering miRNAs, preventing them from interacting with and degrading their target mRNAs, which effectively alters gene expression [15].

Since there are currently no reliable tumor markers or imaging methods that accurately detect early HCC or predict sorafenib and regorafenib effectiveness [16], recent research has started to explore the potential of circulating miRNAs and lncRNAs as tools for detecting HCC, understanding its pathogenesis, and predicting the effectiveness of treatments like sorafenib and regorafenib.

lncRNA KCNQ1 overlapping transcript 1's (*KCNQ1OT1)* role has recently been linked to many cancer types. For instance, it promotes osteosarcoma growth by enhancing aerobic glycolysis via aldolase A and sponging *miR-34c-5p* [17]. In tongue cancer, *KCNQ1OT1* regulates cell proliferation and contributes to resistance by modifying the *miR-211-5p/Ezrin/Fak/Src* signaling pathway [18]. Additionally, *KCNQ1OT1* inhibits the progression of gastric cancer through the *miR-9/*LMX1A axis [19]. In hepatocellular carcinoma (HCC), *KCNQ1OT1* functions as an oncogene by promoting IGF1R expression through sponging *miR-148a-3p* [20]. However, the biological role of *KCNQ1OT1* in cuproptosis and its regulation of DLAT in any form of cancer remain uncertain.

*miR-27b-5p* functions as a tumor suppressor in many cancers by targeting key oncogenic pathways. As, it inhibits proliferation of breast cancer by targeting proliferating cell nuclear antigen (PCNA) [21]. In ovarian cancer, it suppresses cell proliferation, migration and invasion by downregulating the C-X-C motif chemokine ligand 1 (CXCL1) [22]. While in HCC, it mediates the antitumor effects of the natural flavonoid wogonin by targeting tyrosine 3-monooxygenase activation protein zeta (YWHAZ) and modulating the FAK/AKAT/mTOR pathway [23]. However, its role in cuproptosis and the regulation of DLAT remains uninvestigated.

In our study, we seek to clarify the molecular and clinical importance of the *KCNQ1OT1/miR-27b-5p*/DLAT axis in HCC, specifically regarding its role in regulating cuproptosis. Additionally, we will explore how this axis influences HCC pathogenesis, facilitates early diagnosis, aids in therapeutic monitoring, and supports therapeutic stratification.

## Subjects and Methods

### Bioinformatics Analysis

The role of DLAT in HCC was investigated through bioinformatics analysis, as it is one of the proteins associated with the cuproptosis pathway. The Cancer Genome Atlas (TCGA) (https://www.cancer.gov/ccg/access-data) was used to pick expression data to assess DLAT mRNA levels as well as related lncRNAs and miRNAs in both normal tissues and HCC [24]. LncRNAs that might interact with DLAT were predicted using the LncRRI search program (http://rtools.cbrc.jp/LncRRIsearch/) [25]. Then, the best LncRNAs were chosen based on their lowest sum of energy, which indicates a high binding affinity [26]. After that, Starbase software (https://rnasysu.com/encori/) was used to conduct a co-expression analysis between DLAT and chosen lncRNAs [27, 28]. Additionally, Starbase software and miRDB software (https://mirdb.org/) were used to determine the TDMD scores of miRNAs. The TDMD score serves as a quantitative measure to predict the effectiveness of miRNA in binding to and degrading target mRNAs. A higher score suggests a more efficient interaction [29]. Furthermore, LncBase software (https://diana.e-ce.uth.gr/lncbasev3) was used to evaluate the interactions between selected LncRNA and miRNAs with the highest TDMD scores [30, 31]. Finally, Starbase software was utilized once more to perform a co-expression analysis between DLAT and a chosen miRNA.

### Study Subjects

The current study included a total of 180 participants, categorized into 3 groups. Group I (healthy group) consisted of 50 healthy volunteers (33 males and 17 females) with a mean age of 62.3 ± 1.3 years. Group II (untreated HCC patients, Naïve ‘NV’ group) consisted of 50 subjects (36 males and 14 females with a mean age of 62.8 ± 1.2 years). Group III (HCC patients treated group) included 80 patients (59 male and 21 female) with a mean age of 62.9 ± 0.82 years. Group III (Treated group) was further sub-classified into HCC patients treated with sorafenib 40 patients (Sor group, 29 male and 11 female) with a mean age of 65.54 ± 0.89 years and HCC patients treated with regorafenib 40 patients (Rog group, 30 males, 10 female) with a mean age of 60.24 ± 1.57 years.

All participants were matched for age, sex, and ethnicity. Healthy volunteers were recruited from the blood bank of the Cairo University hospitals, while HCC patients were specifically selected from the National Hepatology and Tropical Medicine Research Institute (NHTMRI), Cairo, Egypt.

All patient data were recorded. The inclusion criteria were as follows: (i) patients were diagnosed with HCC according to the guidelines of the American Association for the Study of Liver Diseases (AASLD) [32]; (ii) patients were considered ineligible for surgical intervention; (iii) staging of the patients was determined using the Barcelona Clinic Liver Cancer (BCLC) Staging System, and their liver function, assessed by the Child–Pugh score, was classified as level A or B (≤ 5) [33]; (iv) patients received sorafenib as a first-line treatment between January 1, 2023, and January 1, 2025, and demonstrated a therapeutic response after a minimum of three months of treatment, confirmed by imaging changes on either CT or MRI and/or a decrease in serum α-fetoprotein (AFP) levels compared to previous measurements [34],(v) patients received regorafenib as a second line treatment after showing unresponsive from the sorafenib after a minimum of three months of treatment, confirmed by no imaging changes on either CT or MRI and/or an increase in serum α-fetoprotein (AFP) levels compared to prior clinical measurement [35].

Patients were excluded from the study if they met any of the following criteria: (i) had previously received systemic therapy or (ii) refused sorafenib treatment due to its adverse effects.

All subjects signed a written informed consent. The study was conducted in accordance with the regulations and recommendations of the Declaration of Helsinki and was approved by both the scientific research ethics committee at the faculty of pharmacy, Helwan University (10H2024); the National Hepatology and Tropical Medicine Research Institute in Cairo's (NHTMRI) ethics committee (2425) and the ethical committee of the General Authority for Hospitals and Educational Institutes, Ministry of Health, Egypt (ITH00171).

### Sample Collection

Venous blood samples were collected from all participants and divided into two aliquots. The first aliquot was placed in serum-separating tubes and centrifuged to isolate the serum, which was stored at − 80 °C for the quantification of serum DLAT. The second aliquot was collected 0.5 M EDTA tubes and preserved at − 80 °C for subsequent RNA extraction and reduced glutathione (GSH) levels measurement.

### Biochemical Analysis

#### Determination of Reduced Glutathione (GSH)

GSH levels were measured in the supernatant of lysed blood samples using Bio-diagnostics kits (Egypt)- Catalog NO.: RE10155- in accordance with the manufacturer’s protocol. The quantification of GSH was based on the oxidation of GSH by 5, 5, -dithiobis-2-nitrobenzoic acid (DTNB), resulting in the formation of oxidized glutathione and 5-thio-2-nitrobenzoic acid. The resulting yellow color was measured at an absorbance of 405 nm, which is directly proportional to the concentration of GSH [36]. GSH concentrations in each sample were subsequently calculated using a calibration curve.

#### Determination of Serum DLAT

The serum levels of DLAT were measured using DLAT ELISA kit (Catalog number: CK-bio -26255 provided by Coon Koon Biotech, Shanghai, China) according to the manufacturer's procedure.

### Molecular Analysis

#### RNA Extraction & cDNA Synthesis

Total RNA was extracted from blood samples using the FavorPrep™ Total RNA Extraction Kit (Catalog No. FABRK 001, Geneaid, Taiwan). This RNA was subsequently utilized for cDNA synthesis with the FIREScript™ cDNA Synthesis MIX using oligo and random primers (Catalog No. 06–20-00100, SOLIS BIOBYNE™, Estonia). Quality and quantity of extracted RNA extraction and its cDNA copies were assessed using Nanodrop 2000C® (Thermo Fisher Scientific, Cairo, Egypt).

#### Gene Expression Analysis

The expression levels of *ATP7B, ATP7A, SLC31A1, KCNQ1OT1,* and *miR − 27b − 5p* were determined by using Real-time quantitative PCR (qPCR) with the SolisFAST® SolisGreen® qPCR No-ROX Kits (Catalog No. 28–41-00001, SOLIS BIOBYNE™, Estonia), following the running conditions specified in Table 1. Target gene expression levels were normalized to GAPDH (for protein-coding genes and lncRNAs) or U6 (for miRNAs) and calculated relative to the healthy control group using the 2^ − ^ΔΔCt^ method: ∆CT = CT (Target gene)—CT (Endogenous control), ∆∆CT = ∆CT (Patient)—∆CT (Healthy control), and FC (Fold change) = 2^−∆∆C^. The primer sequences used in the analysis are listed in Table 2.

**Table 1:** Time–temperature conditions for qPCR reaction

Thermal condition	Temperature	Time	No. of cycles
Holding activation step	95 °C	2 min	1 cycle
Denaturation	95 °C	5 s	45 cycles
Annealing and extension	57 °C	20 s	
Melting curve	65- 95 °C	• 90 s pre melt • 5 s each step afterwards	1 cycle

**Table 2:** Primer sequences used in qPCR transcriptomics analysis (5' to 3')

Primers for PCR	Forward sequence	Reverse sequence	Reference
*SLC31A1*	CCAGGACCAAATGGAACCATCC	ACCACCTGGATGATGTGCAGCA	[37]
*ATP7B*	GGACCACAACATCATTCCAGGAC	ATGAGCACGTCCATGTTGGCTG	[38]
*ATP7A*	CCCTCTAGGAACAGCCATAACC	ATACCACAGCCTGGCACAACCT	[38]
*GAPDH*	AAGGTGAAGGTCGGAGTCAA	AATGAAGGGGTCATTGATGG	[39]
*KCNQ1OT1*	GGAGTCTGGAACCTGACATCTG	GTGTCAGGTGATGGAAGGACT	[40]
*miR − 27b − 5p*	GCCAGAGCTTAGCTGATTG	CAGTGCGTGTCGTGGA	[41]
*U6*	GCTTCGGCAGCACATATACTA	CGAATTTGCGTGTCATCCTTG	[39]

### Statistical Analysis

All statistical analyses and graphical presentations were performed using GraphPad Prism software, version 9.0 (La Jolla, CA, USA). The expression levels of *KCNQ1OT1*, *miR-27b-5p*, *SLC31A1, ATP7A, and ATP7B* were calculated using the 2^ − ^ΔΔCt^ method [42] (some representative melt curves were provided in Supplementary section Fig. S5). Sample size estimation and effect size calculation were performed using G*Power software, assuming a medium effect size (f = 0.25), a two-sided significance level of 0.05, and a statistical power of at least 80% [43].

Data normality was assessed using the Kolmogorov–Smirnov, D’Agostino–Pearson, Anderson–Darling, and Shapiro–Wilk tests, which demonstrated a non-parametric distribution. Accordingly, data are presented as median (25th–75th percentile). Comparisons among multiple groups were conducted using the Kruskal–Wallis H test, followed by Dunn’s multiple-comparison post hoc test. Categorical variables were analyzed using the Chi-square (χ^2^) test, and Spearman’s rank correlation analysis was applied to evaluate correlations among the studied parameters.

ΔCt values were used to construct Receiver Operating Characteristic (ROC) curves, and 95% confidence intervals (CI) for the area under the curve (AUC) were calculated to assess the diagnostic and therapeutic monitoring performance of the studied biomarkers. All optimized cut-off value were selected based on the Youden Index (YI) and were provided in Supplementary section. A *p*-value < 0.05 was considered statistically significant.

## Results

### The Demographic and Clinical Characteristics of Study Subjects

All demographic and clinical data of study subjects were shown in Table 3. The study showed that age, sex, smoking status, or family history did not have any significant difference between the studied groups (*p* > 0.05). Additionally, the mean age of the study subjects ranged from 60.24 to 65.54 years, with a male preponderance. HCV was the most prevalent etiology across all HCC groups, while HBV and other causes were infrequent and did not differ significantly among groups. Serum creatinine and hemoglobin levels were similar between the groups, indicating stable renal function and mild or no anemia.

**Table 3:** Demographic, clinical and laboratory characterization of the studied groups

Parameter	Classification	Group I	Group II	Group III		*P* Value
		Healthy group (*n* = 50)	NV group (*n* = 50)	Sor group (*n* = 40)	Rog group (*n* = 40)	
Age (years)		62.31 ± 1.34	65.54 ± 0.89	62.76 ± 1.212	60.24 ± 1.57	NS
Sex	Male	33	36	29	30	NS
	Female	17	14	11	10	
Smoking	Yes	34	35	28	31	NS
	No	16	15	12	9	
Family History	Yes	0	1	0	0	NS
	No	50	49	40	40	
Underlying Causes of HCC	HCV	-	46	38	35	NS
	HBV	-	1	0	4	
	Other	-	3	2	1	
Comorbidity	Yes	14	27	16	16	NS
	No	36	23	24	24	
Tumor Size	Large	-	31	33	39	0.0004^*^
	Small	-	19	7	1	
Metastasis	Yes	-	29	28	34	0.0302^*^
	No	-	21	12	6	
Duration of Treatment	3 months	-	-	9	12	NS
	3–6 months	-	-	12	10	
	6–9 months	-	-	8	8	
	> 9 months	-	-	11	20	
CHILD Score	A5	-	16	19	17	NS
	A6	-	22	19	19	
	B7	-	8	2	4	
	B8	-	4	0	0	
Performance Status	0	-	8	2	9	NS
	1	-	37	26	22	
	2	-	5	11	6	
	3	-	0	1	1	
HB (g/dL)		12.25 (10.70–13.53)	11.65 (10.43–13.35) ^a^	11.80 (11.00–13.80)	13 (12.00–14.00)	0.013
Platelet Count (× 10⁹/L)		225.0 (200.0–282.0) ᵇᶜ^d^	165.5 (125.0–248.3) ᵃ	162 (110.0–205.0) ᵃ	150 (100.0–274.8)^a^	0.0006
Creatinine (mg/dL)		0.9 (0.8–1.1)	0.95 (0.8–1.1)	0.949 (0.79–1.1)	1 (0.825–1.100)	NS
Total Bilirubin (mg/dL)		0.4 (0.3–0.571) ᵇᶜ^d^	1.15 (0.89–1.8) ᵃ	0.89 (0.73–1.363) ᵃ	0.95 (0.8–1.593)^a^	< 0.0001
Albumin (g/dL)		4.071 (3.7–4.814) ᵇᶜ^d^	3.485 (2.993–3.975) ᵃ	3.7 (3.0–4.0) ᵃ	3.61 (3.188–4.1)^a^	< 0.0001
AST (U/L)		20 (16–21.5) ᵇᶜ^d^	53 (34–76) ᵃ	41 (30–66) ᵃ	44 (35.5–65.5)^a^	< 0.0001
ALT (U/L)		22 (14–29) ᵇᶜ^d^	37 (22–55) ᵃ	31 (23–47) ᵃ	35 (25–47.75)^a^	< 0.0001
INR		0.9 (0.85–0.95) ᵇᶜ^d^	1.12 (1.02–1.23) ᵃ	1.07 (1.0–1.21) ᵃ	1.1 (1.0–1.215) ᵃ	< 0.0001
AFP (ng/mL)		1.5 (1–2) ᵇᶜ^d^	75.75 (16.13–1134) ᵃ	259 (21.48–2296) ᵃ	203 (25.25–1520) ᵃ	< 0.0001

Data are presented as median (25th–75th percentile) for non-parametric variables. Numerical data were analyzed using the Kruskal–Walli’s test followed by Dunn’s multiple comparisons test, and significant differences are indicated by letters: a, significant difference from Healthy group; b, significant difference from NV group; c, significant difference from Sor group; d, significant difference from Rog group. Categorical data were analyzed by Chi-square test (X^2^), and significance (*P* < 0.05) is indicated by *. *AST* Aspartate transaminase; *ALT* Alanine transaminase; *HB* Hemoglobin; *INR* International Normalized Ratio for Prothrombin Time; *AFP* Alpha-fetoprotein; *CHILD* Child–Pugh score

In addition, Tumor size was significantly bigger in Rog group (97.5%) than in the Sor (82.5%), and NV (62%) groups (*p* = 0.0004). Likewise, the incidence of metastasis increased across treatment groups, being highest in Rog group in comparison to other groups (*p* = 0.0302).

In comparison to healthy control, all HCC groups had significantly increased AST, ALT, total bilirubin levels and decreased albumin levels particularly in NV group (*p* < 0.0001) which indicated liver dysfunction and tumor burden. Platelet counts were significantly lower in HCC groups compared to healthy groups, especially in the Sor group (*p* = 0.0006).

International Normalized Ratio for Prothrombin Time (INR) levels showed a marked and significant elevation in all HCC groups, indicating coagulopathy, especially noted in the Sor and NV groups (*p* < 0.0001). While Alpha-fetoprotein (AFP) showed a significant elevation in all HCC groups relative to healthy group (*p* < 0.0001). The Sor group exhibited the highest AFP levels followed by the Naïve and Rog groups.

### Bioinformatics Analysis: DLAT-Associated Competing Endogenous RNA (ceRNA) Network in HCC

Based on transcriptomic data and computational tools, DLAT was assessed as a cuproptosis marker in HCC. Expression data obtained from TCGA demonstrated that DLAT mRNA was significantly upregulated in HCC tissue compared to normal liver tissue (Fig. S1 A). The prediction of lncRNAs that interact with DLAT was performed using LncRRI search. Three lncRNAs were identified as the strongest interactors with DLAT based on their lowest sum of interaction energy values: RP11-573D15.8–018 (–5972.38 kcal/mol), *KCNQ1OT1 (*–2072.36 kcal/mol), and RP3-323A16.1–001(–1911.64 kcal/mol).

Following that, co-expression analysis was carried out using Starbase that revealed a statistically significant positive correlation between *KCNQ1OT1* and DLAT (r = 0.065, *P* < 0.001). Additionally, *KCNQ1OT1* was also upregulated in HCC tissue, exhibiting a similar pattern of DLAT (Fig. S1 B, G).

Based on Starbase, 140 miRNAs were predicted to bind to DLAT. From these, 4 miRNAs with highest Target-Directed miRNA degradation Scores (TDMS Scores) were selected for more analysis: *miR-365a-3p* (score = 1.8662), *miR-27b-5p* (score = 1.7646), *miR-1271-5p* (score = 1.1474), and *miR-96-5p* (score = 1.000).

**Fig. 1:**
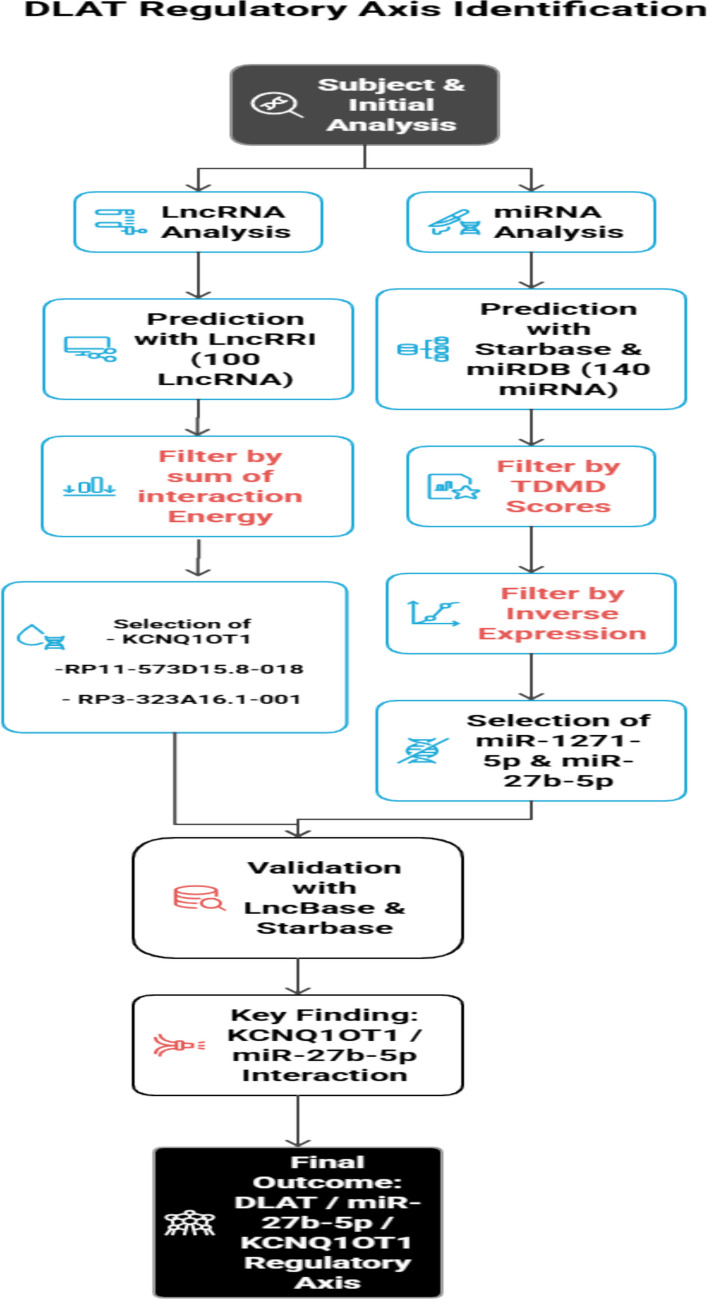
Schematic representation of the bioinformatics analysis regarding DLAT/miR-27b-5p/KCNQ1OT1 axis

Expression profiling using TCGA indicating that miR-96-5p and miR-365a-3p were upregulated, while *miR-1271-5p* and *miR-27b-5p* were downregulated in HCC tissues (Fig. S1 C-F). Since DLAT is upregulated, miRNAs with inverse expression (*miR-1271-5p* and *miR-27b-5p)* were chosen for additional analysis. Potential binding interactions between *KCNQ1OT1* and the two selected miRNAs were validated using both LncBase and Starbase databases. The results showed that *KCNQ1OT1* and *miR-27b-5p had* a TDMD Score of 0.65, which was significantly higher than the score for *KCNQ1OT1–miR-1271-5p* (TDMD Score = 0.004) in liver tissue. These results indicated that DLAT/*miR-27b-5p /KCNQ1OT1* axis may be the potential regulatory axis. All bioinformatics results were shown in supplementary materials (Fig. S1 H-I). Additionally, Fig. 1 illustrates a schematic representation of the bioinformatics analysis involving the DLAT/miR-27b-5p/KCNQ1OT1 axis.

### Serum DLAT Concentration in the Study Subjects

**Fig. 2:**
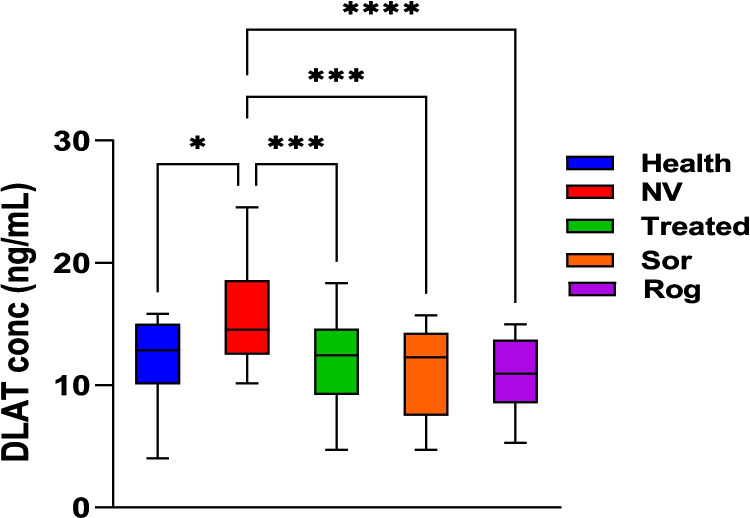
Box-Whisker plots of the serum DLAT level in the studied groups. Data are presented as median (25th–75th percentile, ng/mL), (Healthy, n = 50; NV, *n* = 50; Treated, *n* = 80; Sor, *n* = 40; Rog, *n* = 40). Data were analyzed using the Kruskal–Wallis H test followed by Dunn’s multiple-comparison post hoc test. Statistical significance is indicated as *p < 0.05, ***p < 0.001, ****p < 0.0001

Serum DLAT levels were significantly elevated in the NV group with median was 14.54 ng/mL (12.50–18.59) compared to healthy control group with median 12.85 ng/mL (10.06–15.00; *p* = 0.0392). In addition, serum DLAT level was significantly reduced in both HCC treated group with median was 12.43 ng/mL (9.2–14.6; *p* = 0.0002), Sor group with median was 12.27 ng/mL (7.50–14.27; *p* = 0.0003) and Rog group was 10.93 ng/mL (8.53–13.71; *p* < 0.0001) compared to NV group. Inversely, no significant change in DLAT level was observed between Sor and Rog groups (*p* > 0.05), as illustrated in (Fig. 2).

**Fig. 3:**
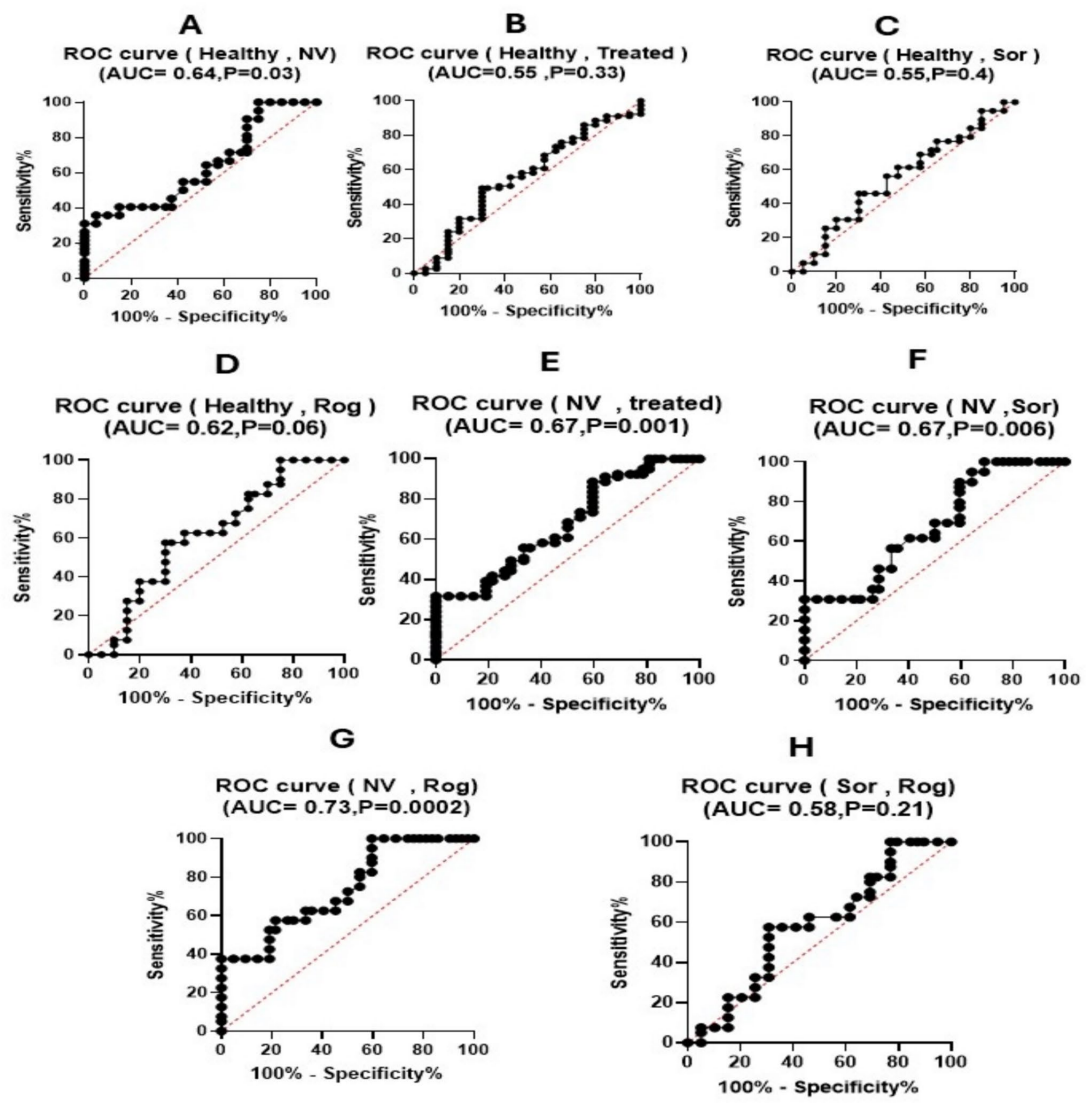
Receiver Operating Characteristic (ROC) Curves for DLAT expression levels in different studied groups. **A** ROC curve for Healthy vs. NV. **B** ROC curve for Healthy vs. Treated. **C** ROC curve for Healthy vs. Sor. **D** ROC curve for Healthy vs. Rog. **E** ROC curve for NV vs. Treated. **F** ROC curve for NV vs. Sor. **G** ROC curve for NV vs. Rog. **H** ROC curve for Sor vs. Rog. A *p*-value < 0.05 is considered statistically significant. Data are expressed as median (25th–75th percentile). The confidence interval (CI) for each curve was calculated at 95%

The diagnostic potential of DLAT was evaluated by ROC curve analysis. ROC analysis showed that DLAT effectively distinguished NV group from healthy controls (AUC = 0.64, *p* = 0.03; cutoff = 13.09; sensitivity = 54.8%, specificity = 57.5%, CI: 0.51–0.76). No significant discrimination was detected between healthy and treated group (AUC = 0.55, *p* = 0.33; cutoff = 12.52; sensitivity = 55.7%, specificity = 57.5%, CI: 0.44–0.67), or Sor group (AUC = 0.55, *p* = 0.40; cutoff = 12.52; sensitivity = 56.4%, specificity = 57.5%, CI: 0.43–0.68), or Rog group (AUC = 0.62, p = 0.06; cutoff = 12.44; sensitivity = 62.5%, specificity = 62.5%, CI: 0.49–0.74) as shown in (Fig. 3, A–D).

In terms of therapeutic monitoring efficacy, DLAT effectively differentiated NV group from treated group (AUC = 0.67, *p* = 0.001; cutoff = 12.66; sensitivity = 58.2%, specificity = 59.5%, CI: 0.58–0.77), Sor group (AUC = 0.67, *p* = 0.006; cutoff = 12.51; sensitivity = 56.4%, specificity = 66.7%, CI: 0.56–0.79), and Rog group (AUC = 0.73, *p* = 0.0002; cutoff = 12.48; sensitivity = 62.5%, specificity = 66.7%, CI: 0.63–0.84). ROC analysis did not indicate a significant difference between the Sor and Rog groups (AUC = 0.58, *p* = 0.21; cutoff = 11.75; sensitivity = 57.5%, specificity = 69.2%, CI: 0.45–0.71) as illustrated in (Fig. 3, E–H).

#### KCNQ1OT1 Expression in the Study Subjects

**Fig. 4:**
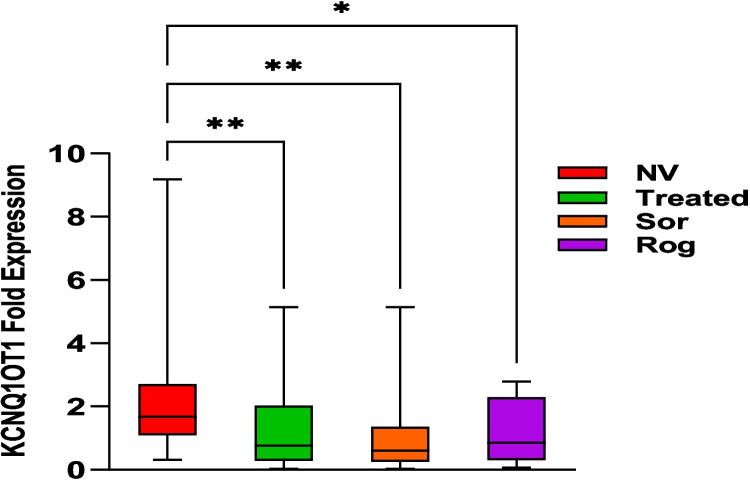
Box-Whisker plots of the median fold expression of *KCNQ1OT1* in the studied groups. Expressed as median fold change from the control group, (NV, *n* = 50; Treated, *n* = 80; Sor, *n* = 40; Rog, *n* = 40) relative to healthy controls is presented as median (25th–75th percentile). Data were analyzed using the Kruskal–Wallis H test followed by Dunn’s multiple-comparison post hoc test. Statistical significance is indicated as *p < 0.05, **p < 0.01

*KCNQ1OT1* is a long non-coding RNA implicated in HCC progression and growth [44]. In the present study, *KCNQ1OT1* expression was significantly elevated in NV group with median fold expression was 1.67 (1.09–2.70) compared to healthy control group. Furthermore, a significant reduction in expression was observed in HCC treated groups. The median fold expression of Treated group was 0.76 (0.29–2.02; *p* = 0.0032), Sor group was 0.61 (0.25–1.35; *p* = 0.0035), and Rog group was 0.85 (0.31–2.29; *p* = 0.05) compared to NV, indicating the strong suppressive effect of both therapies on *KCNQ1OT1* expression. No significant difference in expression level was observed between Sor and Rog groups (*p* > 0.05) as illustrated in (Fig. 4).

**Fig. 5:**
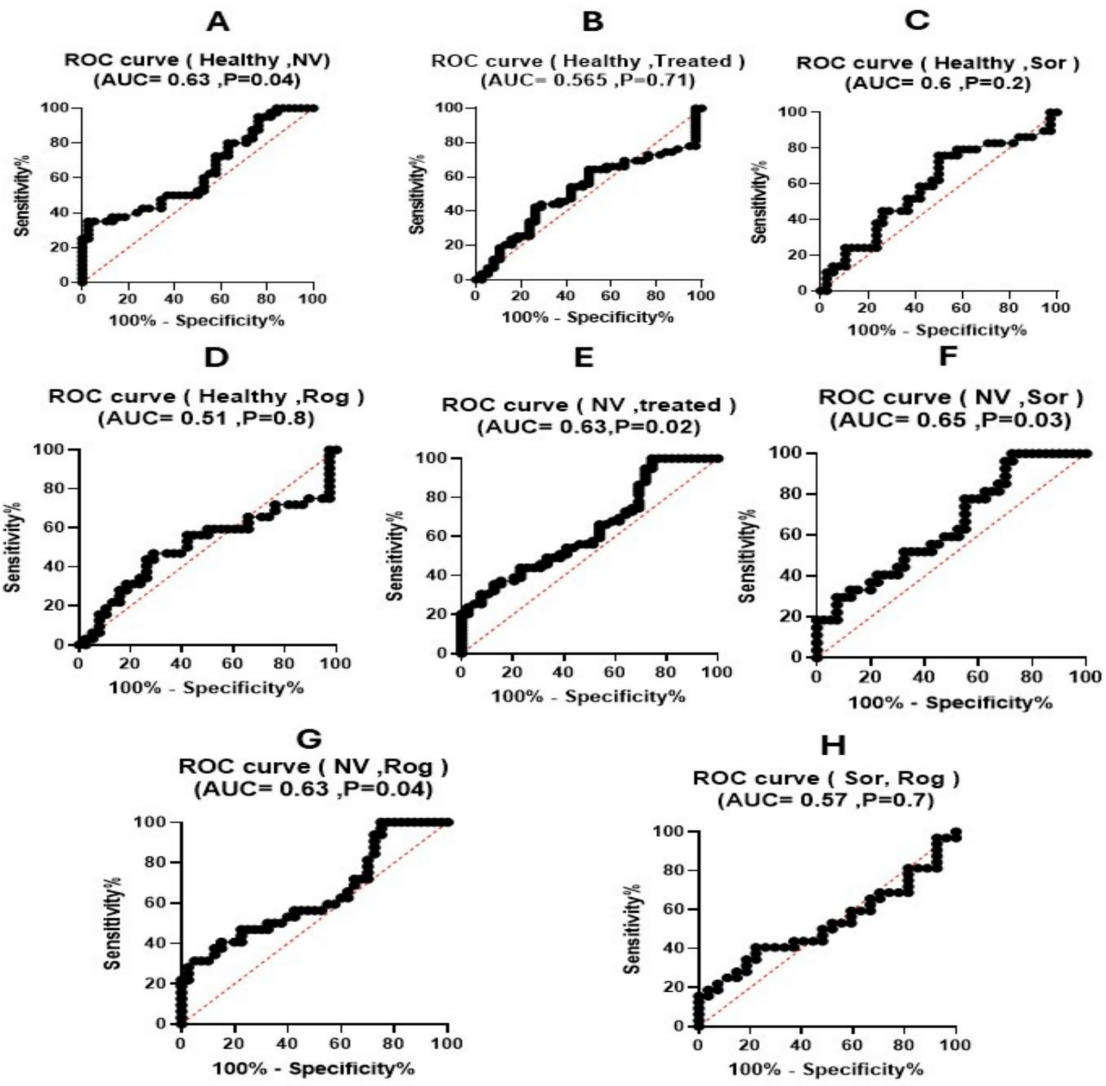
Receiver Operating Characteristic (ROC) Curves for ΔCt value of *KCNQ1OT1* in Different Studied Groups. **A** ROC curve for Healthy vs. NV. **B** ROC curve for Healthy vs. Treated. **C:** ROC curve for Healthy vs. Sor. **D** ROC curve for Healthy vs. Rog. **E** ROC curve for NV vs. Treated. **F** ROC curve for NV vs. Sor. **G:** ROC curve for NV vs. Rog. **H** ROC curve for Sor vs. Rog. A p-value < 0.05 is considered statistically significant. Data are expressed as median (25th–75th percentile). The confidence interval (CI) for each curve was calculated at 95%

The diagnostic potential of *KCNQ1OT1* was evaluated by ROC curve analysis. *KCNQ1OT1* effectively differentiated NV from the healthy group (AUC = 0.63, *p* = 0.04; cutoff = 0.84; sensitivity = 35.0%, specificity = 97.37%, CI: 0.51–0.76). In addition, *KCNQ1OT1* effectively distinguished NV from treated group (AUC = 0.63, *p* = 0.02; cutoff = 1.63; sensitivity = 54.2%, specificity = 59.0%, CI: 0.53–0.75), Sor group (AUC = 0.65, *p* = 0.03; cutoff = 1.63; sensitivity = 55.6%, specificity = 57.5%, CI: 0.52–0.78), and Rog group (AUC = 0.63, *p* = 0.04; cutoff = 1.63; sensitivity = 56.3%, specificity = 57.5%, CI: 0.51–0.77). No significant discrimination was detected between the healthy and treated group (AUC = 0.565, *p* = 0.71; cutoff = 2.07; sensitivity = 42.4%, specificity = 73.7%, CI: 0.40–0.64), neither between the healthy and Sor group (AUC = 0.60, *p* = 0.20; cutoff = 12.52; sensitivity = 56.4%, specificity = 57.5%, CI: 0.45–0.73), nor between the healthy and Rog group (AUC = 0.51, *p* = 0.80; cutoff = 1.63; sensitivity = 56.3%, specificity = 55.3%, CI: 0.37–0.66). Finally, no significant difference was observed between the Sor and Rog groups (AUC = 0.57, *p* = 0.70; cutoff = 1.72; sensitivity = 50.0%, specificity = 51.9%, CI: 0.38–0.68) as illustrated in (Fig. 5, A–H).

#### miR-27b-5p Expression in the Study Subjects

**Fig. 6:**
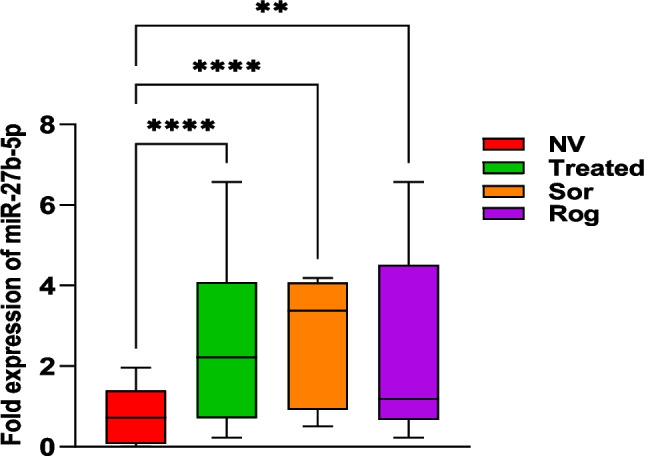
Box-Whisker plots of the median fold expression of *miR-27b-5p* in the studied groups. Expressed as median fold change from the control group. (NV, *n* = 50; Treated, *n* = 80; Sor, *n* = 40; Rog, *n* = 40). Data were presented as median (25th–75th percentile). Data were analyzed using the Kruskal–Wallis H test followed by Dunn’s multiple-comparison post hoc test. Statistical significance is indicated as **p < 0.01, ****p < 0.0001

The expression level of *miR-27b-5p* decreased significantly in NV group with median fold expression was 0.73 (0.08–1.40) compared to healthy group. On the other hand, HCC treated groups exhibited a significant increase in expression of *miR-27b-5p,* indicating that both treatments effectively restored *miR-27b-5p* levels. Treated group showed median fold expression was 2.22 (0.71–4.08; *p* < 0.0001), Sor group was 3.38 (0.91–4.08; *p* < 0.0001), and Rog group was 1.19 (0.67–4.52; *p* = 0.01) compared to NV group (Fig. 6).

**Fig. 7:**
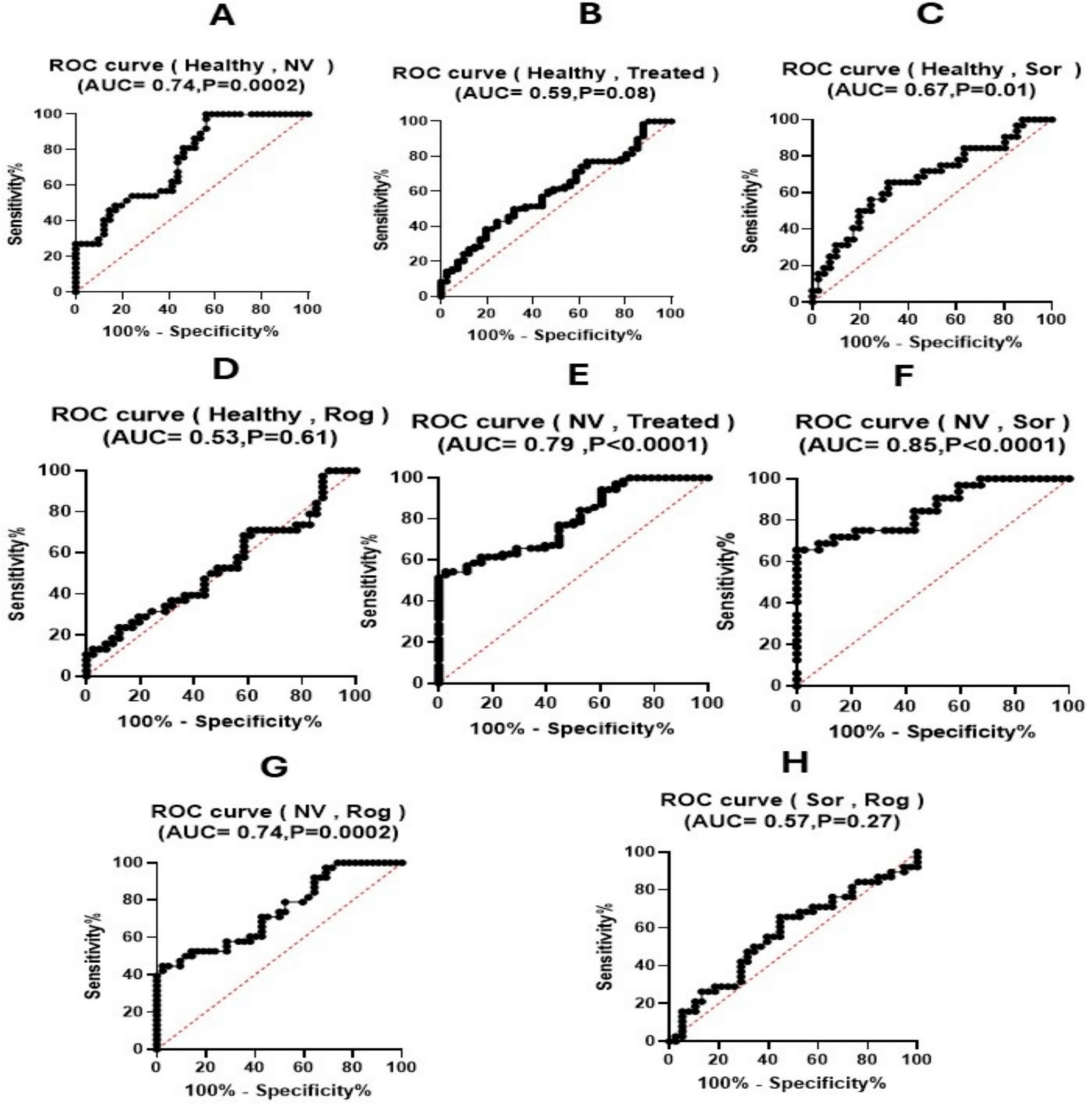
Receiver Operating Characteristic (ROC) Curves for ΔCt values of *miR-27b-5p in* different studied groups. **A** ROC curve for Healthy vs. NV. **B** ROC curve for Healthy vs. Treated. **C:** ROC curve for Healthy vs. Sor. **D** ROC curve for Healthy vs. Rog. **E** ROC curve for NV vs. Treated. **F:** ROC curve for NV vs. Sor. **G:** ROC curve for NV vs. Rog. **H:** ROC curve for Sor vs. Rog. The confidence interval (CI) for each curve was calculated at 95%

Regarding its diagnostic performance, *miR-27b-5p* significantly distinguished NV group from healthy group (AUC = 0.74, *p* = 0.0002; cutoff = 12.72; sensitivity = 54.1%, specificity = 73.2%, CI: 0.63–0.85) and differentiated Sor group from healthy group (AUC = 0.67, *p* = 0.01; cutoff ≈10.22; sensitivity = 65.6%, specificity = 68.3%, CI: 0.55–0.80). In contrast, no significant discrimination was observed between Rog and healthy groups (AUC = 0.53, p = 0.61; cutoff = 12.43; sensitivity = 71.1%, specificity = 39.0%, CI: 0.40–0.66), as shown in (Fig. 7 A-D).

For therapeutic monitoring, *miR-27b-5p* significantly differentiated Treated, Sor, and Rog groups from NV group (Treated: AUC = 0.79, *p* < 0.0001; cutoff ≈11.76; sensitivity = 64.3%, specificity = 71.1%, CI: 0.71–0.88; Sor: AUC = 0.85, *p* < 0.0001; cutoff = 11.66; sensitivity = 75.0%, specificity = 78.4%, CI: 0.77–0.95; Rog: AUC = 0.74, *p* = 0.0002; cutoff ≈12.02; sensitivity = 61.9%, specificity = 60.5%, CI: 0.63–0.85). In contrast, Sor and Rog groups were not significantly distinguished (AUC = 0.57, *p* = 0.27; cutoff = 10.20; sensitivity = 65.8%, specificity = 55.3%, CI: 0.44–0.70) as in (Fig. 7 E–H).

#### Expression of Cuproptosis-related Genes in the Study Subjects

To examine the involvement of cuproptosis in HCC, the expression levels of cuproptosis-related genes (*ATP7A, ATP7B, SLC31A1*) and key copper-binding antioxidant molecule, reduced glutathione (GSH) were evaluated across the examined groups.

**Fig. 8:**
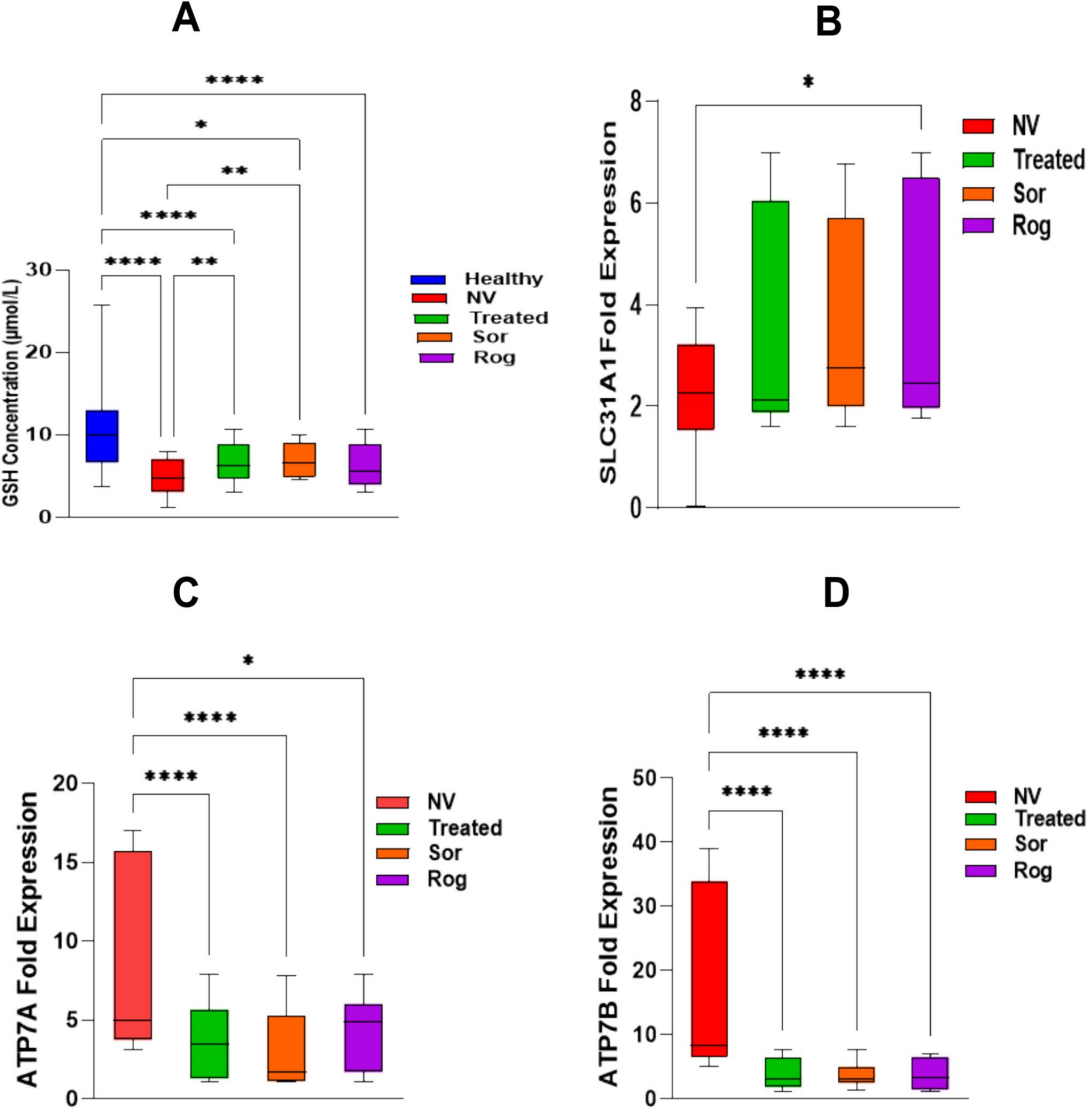
Box-Whisker plots of the median serum glutathione (GSH) level and expression of cuproptosis related genes in the study subjects, expressed as median fold change from the control group. A: Serum GSH concentration in different groups (Healthy, *n* = 50; NV, *n* = 50; Treated, *n* = 80; Sor, *n* = 40; Rog, *n* = 40; µmol/L). B: Fold expression of SLC31A1. C: Fold expression of ATP7A. D: Fold expression of ATP7B. Data were analyzed using the Kruskal–Wallis H test followed by Dunn’s multiple-comparison post hoc test. Statistical significance is indicated as *p < 0.05, **p < 0.01, ****p < 0.0001

Regarding GSH levels, it was found that GSH levels decreased significantly in all studied groups compared to healthy control. The NV group demonstrated median GSH levels (4.78 (3.08–7.10) µmol/L, *p* < 0.0001), Treated group (6.36 (4.77–8.87) µmol/L, *p* < 0.0001), Sor group (6.60 (4.90–9.07) µmol/L, *p* = 0.016), and Rog group (5.54 (3.98–8.87) µmol/L, *p* < 0.0001) compared to healthy group (9.93 (6.68–13.03) µmol/L). Furthermore, HCC treated groups showed elevated GSH levels compared to NV group (Treated: *p* = 0.0085; Sor: *p* = 0.0077; Rog: *p* = 0.211) as shown in (Fig. 8A).

The fold expression of *SLC31A1* was considerably elevated in all HCC groups, as NV group showed median fold expression as 2.3 (1.5–3.21)-folds, treated group 2.14 (1.88–6.05)-folds, Sor group 2.76 (1.99–5.70)-folds, and Rog group 2.46 (1.96–6.50)-folds compared to healthy group. Additionally, HCC treated with Rog demonstrated significantly elevated median fold expression compared to NV group (*p* = 0.014). No significant difference was observed between other groups (*p* > 0.05) as illustrated in (Fig. 8B).

Regarding *ATP7A*, the expression level was significantly increased in all HCC studied groups compared to healthy groups. However, these expression levels notably decreased in both treatment groups: the median fold change was 3.42 (1.29–5.66) for the treated group (*p* < 0.0001), 1.70 (1.12–5.24) for the Sor group (*p* < 0.0001), and 4.90 (1.69–6.01) for the Rog group (*p* = 0.0288) compared to median fold change 5.01 (3.72–15.73) in NV group. No significant difference was observed between Sor and Rog groups (*p* = 0.37). (Fig. 8C).

The same scenario occurred in *ATP7B*, the expression level was significantly greater in HCC groups compared to healthy. But after treatment with both Sorafenib and Regorafenib, its expression levels decreased significantly compared to NV group (*p* < 0.0001). The median fold expression of Treated group was 2.93 (1.74–6.35), Sor group was 2.93 (2.39–4.99), and Rog group was 3.27 (1.39–6.48) compared to NV group (8.27, 6.46–33.89). Nevertheless, there was no statistically significant difference between Rog and Sor groups (*p* > 0.05) (Fig. 8D).

### Correlation Analysis of KCNQ1OT1, miR-27b-5p and DLAT in Study Subjects

Spearman correlation analysis was performed to assess correlations between *KCNQ1OT1, miR-27b-5p*, and DLAT expression levels across different studied groups. *KCNQ1OT1* showed a significant negative correlation with *miR-27b-5p* in NV group (r = –0.40, *p* = 0.02), Sor group (r = –0.46, *p* = 0.002), Rog group (r = –0.58, *p* = 0.009) and treated group (r = –0.43, *p* = 0.0004). Regarding *KCNQ1OT1*and DLAT correlation, a significant positive correlation was found in Sor group (r = 0.38, *p* = 0.02), Rog group (r = 0.33, *p* = 0.03) and treated group (r = 0.28, *p* = 0.01). However, this correlation was not significant in NV group (r = 0.21, *p* = 0.22).

In contrast, *miR-27b-5p* significantly demonstrated a negative correlation with DLAT in NV group (r = –0.34, *p* = 0.04), Sor group (r = –0.33, *p* = 0.03), Rog group (r = –0.34, *p* = 0.03), and treated group (r = –0.33,*p* = 0.002), as illustrated in Table 4, (additional data are given in Fig. S2, 3, 4 A-D).

**Table 4:** Correlation analysis between *KCNQ1OT1*, *miR-27b-5p* and DLAT expression in different groups

Correlation pair	*KCNQ1OT1 vs miR-27b-5p*		*KCNQ1OT1* vs DLAT		*miR-27b-5p* vs DLAT	
Groups	r value	*p* value	r value	*p* value	r value	*p* value
NV	–0.4	0.02*	0.21	0.22	–0.34	0.04*
Sor	–0.46	0.002**	0.38	0.02*	–0.33	0.03*
Rog	–0.58	0.009**	0.33	0.03*	–0.34	0.03*
Treated	–0.43	0.0004***	0.28	0.01*	–0.33	0.002**

The Spearman correlation coefficient (r). *, **, *** *p* < 0.05, *p* < 0.01, *p* < 0.001 respectively

Additionally, the analysis examined varying correlation patterns among *KCNQ1OT1, miR-27b-5p*, DLAT, and various demographic and clinical data across the studied groups to gain insights into their potential roles in HCC progression and treatment response.

In the NV group, DLAT showed a significant a negative correlation with creatinine (r = –0.41, *p* = 0.014). Additionally, there was a positive correlation between the CHILD score and *KCNQ1OT1* expression (r = 0.34, *p* = 0.03). In the Sor group, DLAT negatively correlated with AST (r = –0.34, *p* = 0.04), while *KCNQ1OT1* positively correlated with albumin (r = 0.48, *p* = 0.009).

In the Rog group, DLAT was positively correlated with bilirubin (r = 0.36, *p* = 0.04). Conversely, there was a negative correlation between platelet count and *KCNQ1OT1*(r = –0.40, *p* = 0.03). Furthermore*, miR-27b-5p* positively correlated with AFP (r = 0.35, *p* = 0.03) (additional data are provided in Table S1).

## Discussion

Hepatocellular carcinoma (HCC) remains as significant worldwide healthy concern due to its delayed diagnosis, elevated recurrence rates, and restricted efficacy of existing treatments [2]. Recently,attention has been shifted to a new cell death mechanism, such as cuproptosis,which is closely linked to mitochondrial metabolism and intercellular copper homeostasis [6]. In this study the *KCNQ1OT1/miR-27b-5p*/DLAT axis was examined as a potential regulatory pathway controlling cuproptosis in HCC. We aimed to determine the diagnostic, prognostic, and therapeutic significance of this axis by integrating transcriptomic, clinical, and treatment-related data in regulating cuproptosis in HCC. We also explore how both sorafenib and regorafenib may differentially effect cuproptotic signaling and disease progression. Our findings offer novel insights into HCC biology and propose molecular targets for improving therapy monitoring.

Patients’ demographic and clinical data showed that male are predominant, with the typical age range being 60 to 66 years, which reflects the epidemiology of HCC [45]. Hepatitis C virus (HCV) was the predominant underlying etiology, particularly in Egypt which has a high HCV prevalence [46]. Clinically, the Rog group exhibited more advanced tumor burden, with significantly large tumor sizes and higher metastasis rates because regorafenib is used as 2nd line treatment after sorafenib resistance in more progressed patients [47].

To investigate novel biomarkers and understand their role in cuproptosis and HCC pathogenesis, our study used transcriptomics computational bioinformatics analysis to find regulatory biomarkers and their competing endogenous RNA (ceRNA) network. DLAT was selected for further investigation for many reasons. DLAT has a vital role in cell metabolism and cellular homeostasis [8]. Normally in a healthy physiological state, it is a component of the mitochondrial pyruvate dehydrogenase complex (PDC), where it assists in transferring an acetyl group from pyruvate to coenzyme A. This links glycolysis to the tricarboxylic acid (TCA) cycle [48]. But the dysregulation of DLAT has been linked to metabolic reprogramming that moves cells from oxidative phosphorylation to aerobic glycolysis [49].This change leads to increase cancer growth as observed in HCC, gastric, lung, and esophageal cancers [50]. In addition to its metabolic role, DLAT was the central regulator of cuproptosis, where excessive copper directly binds to lipoylated DLAT, leading to aberrant protein aggregation, and proteotoxic stress. Furthermore, Huang and his colleagues showed that DLAT is a superior biomarker compared to AFP with high sensitivity and specificity, especially in AFP-negative HCC cases. This indicates that DLAT may serve as a promising diagnostic biomarker for diagnosing HCC [51]. This dual role in metabolism and cuproptosis makes it a potential biomarker [52] and therapeutic target [53, 54].

The Cancer Genome Atlas (TCGA) data analysis exhibited that DLAT was significantly upregulated in HCC tissues in relation to normal liver tissue, indicating a potential oncogenic role and a decrease in cuproptosis in HCC, consistent with results from previous research [51, 55–57]. LncRNA–mRNA interaction prediction identified many lncRNA regulators of DLAT, among which *KCNQ1OT1* showed a strong interaction based on the low interaction energy value. Furthermore, co-expression analysis showed a significant positive correlation between *KCNQ1OT1* and DLAT expression. Notably, *KCNQ1OT1* was significantly upregulated in HCC tissues, mirroring DLAT expression, indicating a possible regulatory pathway.

Further analysis of miRNA–mRNA interaction revealed that *miR-27b-5p is* significantly downregulated in HCC and has an inverse correlation with DLAT expression. Moreover, *miR-27b-5p demonstrated* an effective interaction with DLAT, which was shown by a high TDMD score*. KCNQ1OT1* exhibited a notable binding affinity for *miR-27b-5p.* The results indicate that *KCNQ1OT1* may sequester *miR-27b-5p,* hence inhibiting its ability to downregulate DLAT.

To validate the clinical significance of DLAT, its serum concentration was analyzed. Serum DLAT levels were significantly increased in untreated HCC patients (NV group) compared to healthy and treated groups. This may be due to DLAT overexpression which promotes HCC cell proliferation, migration and invasion [58]. In addition, DLAT can increase glucose transporter 1 **(**GLUT1) expression, promoting glycolysis, which induce tumor invasion and metastasis [56]. DLAT overexpression results were supported by ROC analysis, where DLAT significantly differentiate NV patients from healthy groups. Also, it is correlated with metastasis and Child–Pugh score which further supports its role in HCC. In the same way, these findings highlight the role of DLAT as diagnostic biomarker. In addition, the results of ROC curves showed no significant difference between healthy and treated groups. DLAT can differentiate NV from treated groups (Sor and Rog groups), which support that DLAT can be used as potential therapeutic monitoring biomarker.

Notably, patients who were treated with sorafenib demonstrated the lower DLAT levels than NV group, likely due to the drug's dual role in mitochondrial integrity and energy metabolism. Sorafenib directly affects the PDC,where DLAT serves as a component of PDC, by altering mitochondrial morphology and inhibiting oxidative phosphorylation [59]. Our findings align with the hypothesis that HCC cells with high DLAT expression are predicted to be more sensitive to sorafenib treatment, so reduction in DLAT expression after treatment in our study may suggest successful drug targeting, supporting a potential as therapeutic monitoring biomarker [60, 61].

In the current study, *KCNQ1OT1* expression was the highest in NV group, while Sor and Rog groups showed the lowest expression levels among all groups, reflecting the sorafenib’s and regorafenib’s inhibitory effect on the oncogenic *KCNQ1OT1. KCNQ1OT1* facilitates HCC progression, and its knockdown has been shown to induce apoptosis and reduce cell proliferation, migration and invasion [62]. In addition, Zhang et al., showed that *KCNQ1OT1* knockdown significantly enhances sorafenib sensitivity by targeting miR-506, promoting apoptosis, and suppressing metastasis in sorafenib-resistant HCC cells [63].

The upregulation of *KCNQ1OT1* in NV group was supported by ROC analysis, where it significantly differentiates NV group from healthy group. Furthermore, Regorafenib significantly diminished *KCNQ1OT1* expression compared to the NV group. The alteration in expression was reflected in ROC analysis, where *KCNQ1OT1* differentiated the NV group from treated group, particularly, within the sorafenib and regorafenib subgroups. On the other hand, no significant difference was observed between Sor and Rog subgroups with healthy group, indicating partial normalization of expression after treatment. These results highlight the potential role of *KCNQ1OT1*as diagnostic, therapeutic monitoring and therapeutic stratification biomarker. This aligns with prior research that identified that *KCNQ1OT1* is prognostic biomarker in colorectal cancer [64] and sepsis [65], as well as diagnostic biomarker in glaucoma [66] and breast cancer risk assessment [67].

In context of the previous expression of DLAT and *KCNQ1OT1.* We further explored the *miR-27b-5p* expression profile. *miR-27b-5p* was significantly downregulated in NV group compared to healthy group. This reduction corresponds with its role as tumor suppressor. *miR-27b-5p* has been documented to inhibit cell growth, migration and invasion while promoting apoptosis via regulating Bax and Bcl-2 [68]. In HCC, it targets Fbxw7, a crucial tumor suppressor, and its downregulation is linked to increase tumor growth and diminished apoptosis [69]. In ovarian and gastric cancers, it suppresses tumor progression by targeting genes like CXCL1 [70, 71]. In addition, *miR-27b-5p expression* was elevated post treatment with both Sor and Rog with the highest expression observed in Sor group. These findings are consistent with TCGA data revealing that HCC patients who are treated by sorafenib with high miR-27b expression are associated with longer survival [72]. Wenjing et al. showed that miR-27b increased sorafenib efficacy via modulating the CCNG1–p53 pathway and decreasing CYP1B1-mediated drug detoxification [73].

These expression patterns were supported by ROC analysis. *miR-27b-5p identified* NV patients from healthy, treated, Rog and Sor groups. Additionally, no significant difference was shown between Sor and Rog subgroups, reinforcing *miR-27b-5p* role in diagnosis, treatment monitoring and guiding suggesting post-treatment normalization. The results are supported by previous literature that indicates *miR-27b-5p* as prognostic marker in ovarian cancer [74], as well as a biomarker for disease progression in gastric cancer [75].

Correlation analysis was then performed to further explore the functional interaction and regulatory relationships within the *KCNQ1OT1* /*miR-27b-5p* /DLAT axis. *KCNQ1OT1* showed a negative correlation with *miR-27b-5p across* all groups, suggesting a potential role as a molecular sponge, which should be confirmed by further mechanistic studies. A significant positive correlation between *KCNQ1OT1* and DLAT was observed in healthy and all treated groups, but not in the NV group, indicating disruption of this ceRNA regulatory axis in active disease. Likewise, *miR-27b-5p exhibited* a negative correlation with DLAT only in the healthy and treated groups, highlighting a loss of miRNA-mediated suppression in the NV group. These findings confirm the molecular and clinical significance of the *KCNQ1OT1/miR-27b-5p* /DLAT axis in the regulation of cuproptosis and HCC progression and its responsiveness to therapy.

To further clarify the role of cuproptosis in HCC, the study analyzes the key cuproptosis related- genes *ATP7A, ATP7B, SLC31A1*, and GSH, uncovering the copper dynamics and redox states associated with disease progression and treatment response.

In the healthy group, a homeostatic copper state was observed by raising expression of *SLC31A1* (copper import), diminishing the *ATP7A* and *ATP7B* (copper exporters), increasing GSH levels and reducing circulating DLAT compared to NV group. This physiological profile reflects a normal balanced state in which the copper is efficiently utilized in mitochondrial respiration via activated cytochrome c oxidase and SOD1 enzymes, antioxidant defense without inducing cytotoxicity, aberrant protein aggregation or cuproptosis stress [76].

Conversely, the NV group demonstrated a cuproptosis-evasive molecular profile. Elevated DLAT levels may act as sequestering of intercellular copper and inhibiting its binding to lipoylated proteins, thus reducing the harmful oligomerization and proteotoxic stress that typically trigger cuproptosis [77]. This was also coupled with increasing the *SLC31A1* expression, inducing copper import, and an elevation in *ATP7A* and *ATP7B* expression, which promotes copper efflux compared to the health group. These results show a compensating mechanism which stops copper from accumulation. Even with these changes, significantly reduced GSH levels indicated copper-induced oxidative stress, but the threshold for initiating cuproptotic cell death was not reached but rather may stimulate HCC progression by activating oncogenic signaling pathways and supporting tumor cell growth [78].

Sorafenib and regorafenib treatments induced alterations consistent with the activation of cuproptosis. *ATP7A & ATP7B* expressions were significantly reduced, while *SLC31A1* expression increased compared to NV group. These alterations indicate an augmented copper influx, diminished efflux, and elevated oxidative stress, with the lowering of DLAT likely reflecting its engagement in copper-related processes rather than direct oligomerization—a step associated with cuproptosis [76]. Increasing GSH levels in treated groups compared to NV may be a natural compensatory mechanism that cells use to combat excess Cu [78]. Wang et al. showed that sorafenib inhibits the degradation of FDX1 causing protein lipoylation and possible activation of cuproptosis in HCC [13]. These findings align with evidence that copper enhances regorafenib delivery by binding to heavy -chain ferritin, simultaneously disturbing cellular copper homeostasis and inducing cuproptosis [79].

This study has several limitations. First, intracellular copper content and DLAT oligomerization, which are hallmark events of cuproptosis, were not directly measured. Second, the study relied primarily on serum biomarkers and gene expression analysis without in vitro functional validation or mechanistic assays. Third, while changes in copper transporters, DLAT, and GSH levels were interpreted in the context of cuproptosis based on prior literature, these associations remain indirect. Future studies incorporating copper quantification, DLAT aggregation assays, and functional cell-based models are required to confirm the mechanistic involvement of cuproptosis in sorafenib and regorafenib response.

In conclusion, our findings highlight the *KCNQ1OT1*/*miR-27b-5p*/DLAT axis as a crucial and clinically relevant biomarker network in HCC. This novel axis offers opportunities for improving early diagnosis and assessing therapeutic efficacy, particularly in relation to cuproptosis regulation, HCC progression, and the cuproptosis activation effects of sorafenib and regorafenib.

## Supplementary Information

Below is the link to the electronic supplementary material.

Supplementary file1 (DOCX 4495 KB)

## Data Availability

The data analyzed or employed in the present work can be obtained from this work or from supplementary information. Additional raw data may be available from the corresponding author upon request.

## Abbreviations

*ATP7A*: ATPase Copper Transporting Alpha
*ATP7B*: ATPase Copper Transporting Beta
DLAT: Dihydrolipoamide S-acetyltransferase
INR: International Normalized Ratio for Prothrombin Time
*KCNQ1OT1*: KCNQ1 overlapping transcript 1
*SLC31A1*: Solute Carrier Family 31 Member 1
TCGA: The Cancer Genome Atlas
TDMD: Target-Directed miRNA Degradation
