# Supplementary material for: Role of KCNQ1OT1 / miR-27b-5p in Modulating Dihydrolipoamide S-acetyltransferase (DLAT): Insights into Cuproptosis in Hepatocellular carcinoma
Source: Biol Trace Elem Res. 2026 Feb 18;204(7):4825–43. doi: 10.1007/s12011-026-04999-6 (PMC13319179; doi:10.1007/s12011-026-04999-6)
Supplement: Supplementary file 1 — Supplementary file1 (DOCX 4495 KB) [file 12011_2026_4999_MOESM1_ESM.docx]

**Biological Trace Element Research journal**

**Role of KCNQ1OT1 / miR-27b-5p in Modulating DLAT: Insights into Cuproptosis in Hepatocellular carcinoma**

**Ahmed S. Elkateb^1^, Heba Taha^1^, Tamer Abou Elela^2^, Rehab Ahmed Abdel- Hamid^2^, Sahar A. Ali^1^, Hanaa B. Atya^1^**

**^1^** Biochemistry and Molecular Biology Department, Faculty of Pharmacy, Helwan University, P.O. Box 11795, Cairo, Egypt.

**^2^** Department of Hepatology & Gastroenterology, National Hepatology and Tropical Medicine Research Institute (NHTMRI), Cairo, Egypt.

^*^ Correspondence: Dr. Hanaa B. Atya, [hanaa.atya@pharm.helwan.edu.eg](mailto:hanaa.atya@pharm.helwan.edu.eg), Tel: +201143459600.

**Supplementary informations**


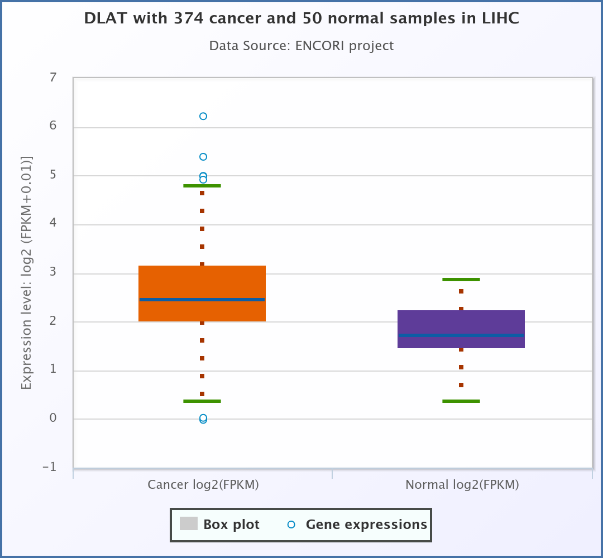

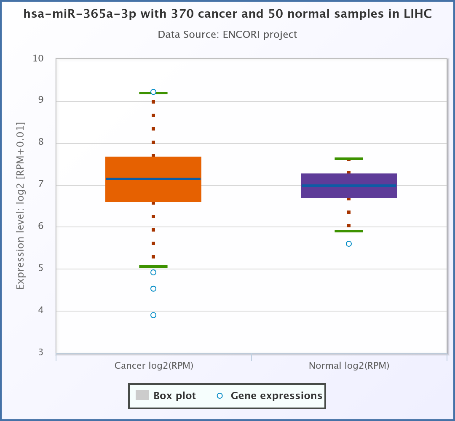

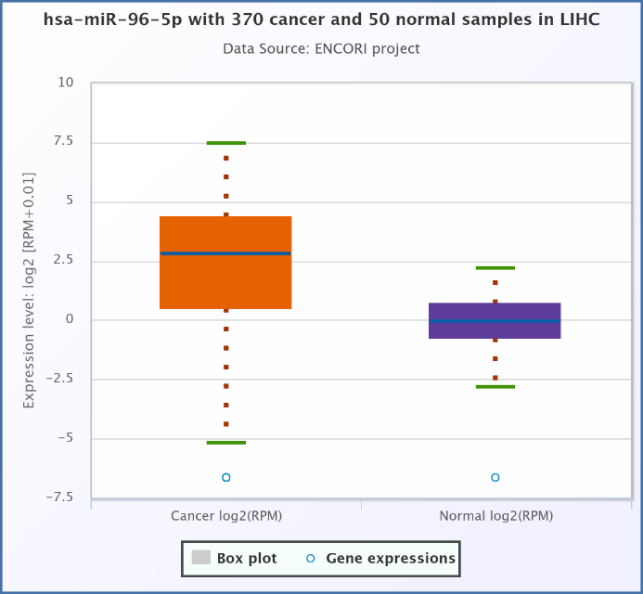

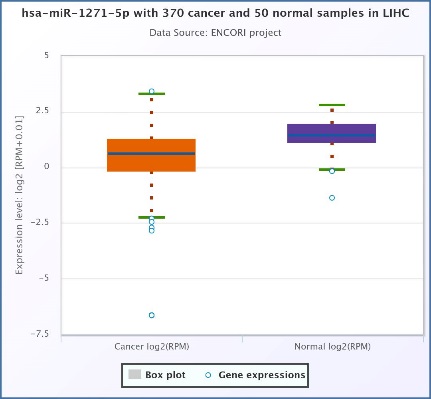

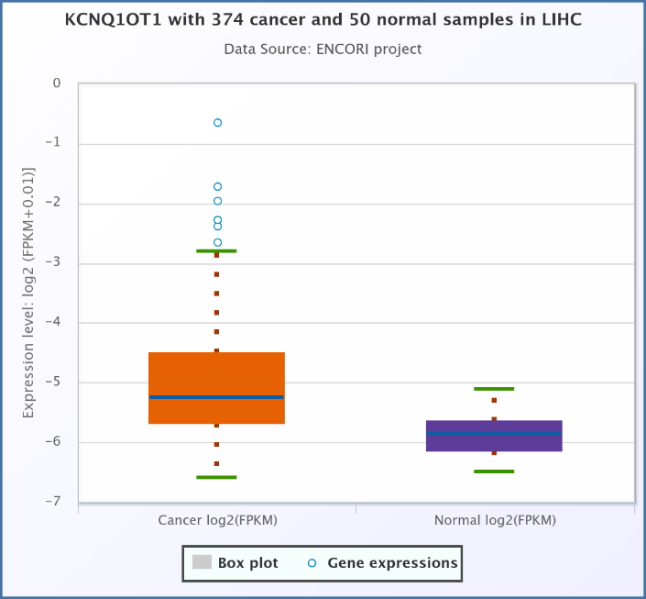

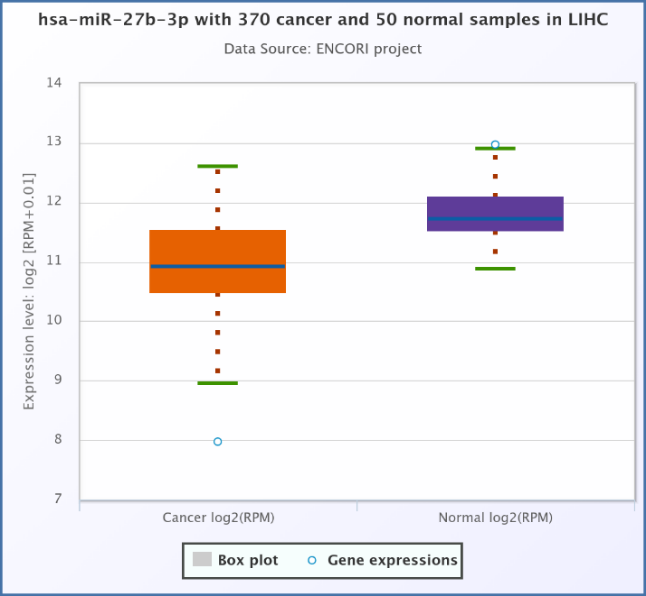


**A**

**B**

**E**

**F**

**5p**

**C**

**D**

**H**

**G**


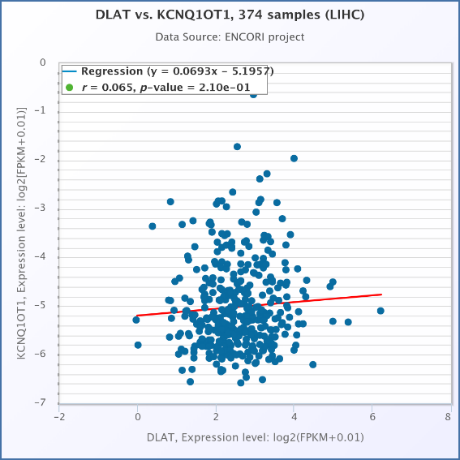

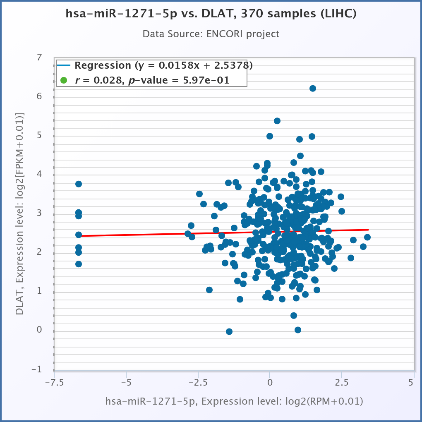

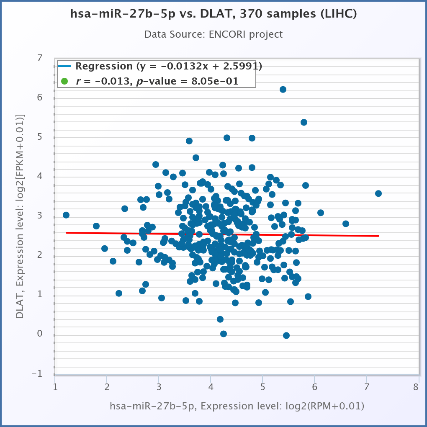


**I**

**Fig. S1:** **Bioinformatic analysis of the KCNQ1OT1/miR-27b-5p/DLAT axis in hepatocellular carcinoma (HCC).**

**A:** Expression levels of DLAT in hepatocellular carcinoma (HCC) tissues compared to normal liver tissues based on TCGA results.

**B:** Expression of long noncoding RNA KCNQ1OT1 in hepatocellular carcinoma (HCC) tissues compared to normal liver tissues based on TCGA results.

**C:** Expression of miR-365-5p in hepatocellular carcinoma (HCC) tissues compared to normal liver tissues based on TCGA results.

**D:** Expression of miR-27b-5p in hepatocellular carcinoma (HCC) tissues compared to normal liver tissues based on TCGA results.

**E:** Expression of miR-96-5p in hepatocellular carcinoma (HCC) tissues compared to normal liver tissues based on TCGA results.

**F:** Expression of miR-1271-5p in hepatocellular carcinoma (HCC) tissues compared to normal liver tissues based on TCGA results.

**G:** Correlation between KCNQ1OT1 and DLAT using both LncBase and Starbase databases.

**H:** Correlation between miR-27b-5p and DLAT using both LncBase and Starbase databases.

**I:** Correlation between miR-1271-5p and DLAT using both LncBase and Starbase databases.


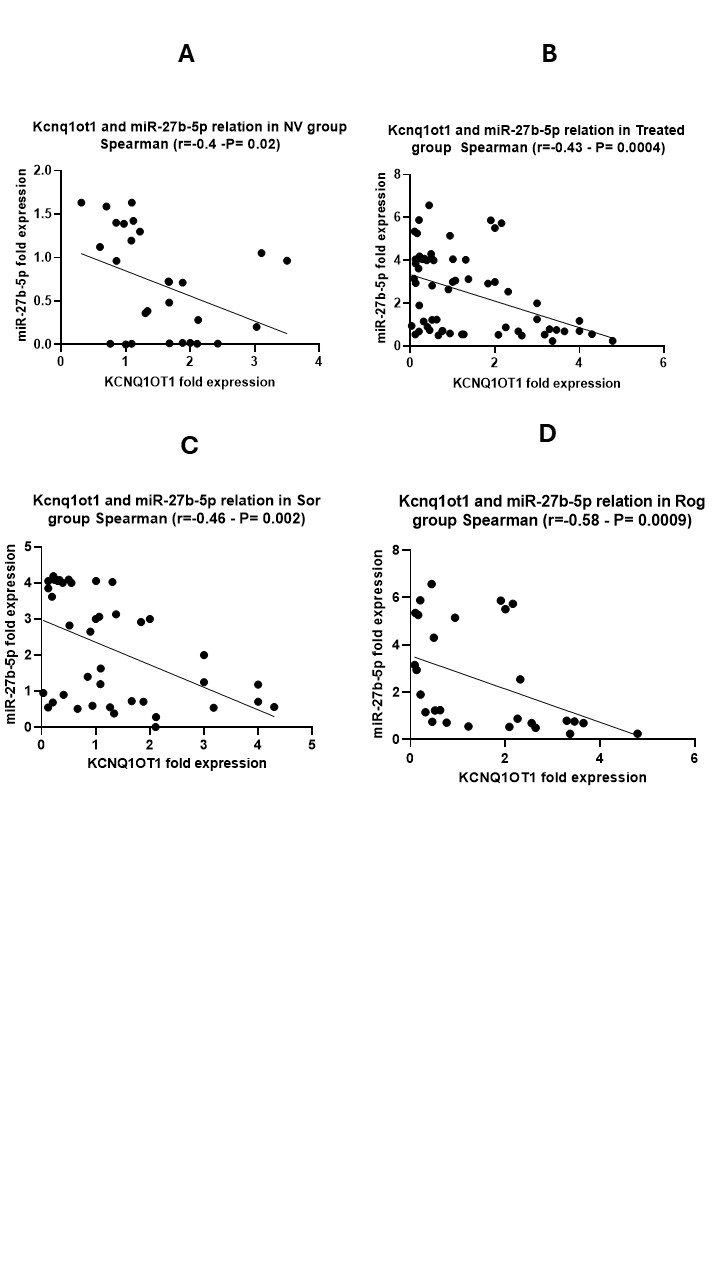


**Fig. S2:** **Correlation analysis Between KCNQ1OT1 and miR-27b-5p Fold Expression in Different Groups.**

Spearman correlation between miR-27b-5p fold expression and KCNQ1OT1 fold expression in the following groups: **A:** NV group (untreated HCC patients), **B:** Treated group (HCC patients treated by sorafenib or regorafenib), **C:** Sor group (HCC patients treated with sorafenib), and **D:** Rog group (HCC patients treated with regorafenib). The Spearman correlation coefficient (r) and p-value are indicated in each panel. A p-value P< 0.05 is considered significant.


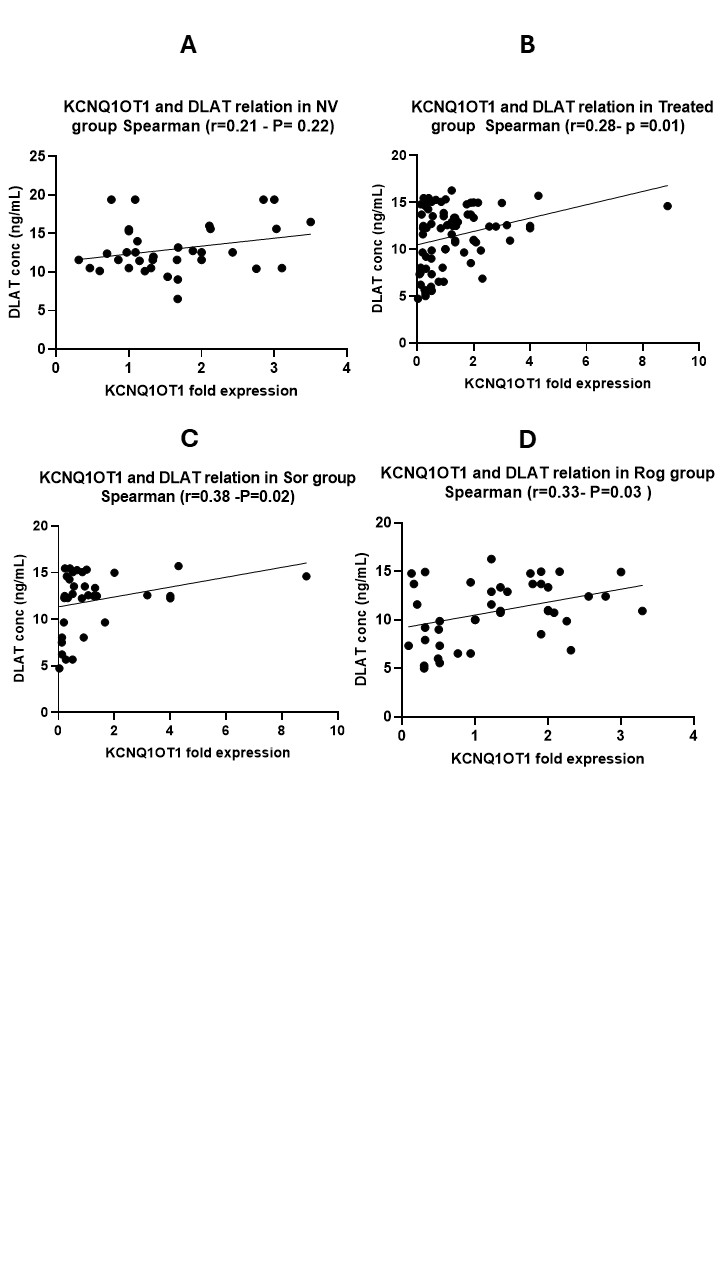


**Fig. S3:** **Correlation analysis Between KCNQ1OT1 Fold Expression and DLAT Concentration in Different Groups.** Spearman correlation analysis between KCNQ1OT1 fold expression and DLAT conc is presented for the following groups: **A:** NV group (untreated HCC patients), **B:** Treated group (HCC patients treated by sorafenib or regorafenib), **C:** Sor group (HCC patients treated with sorafenib), and **D:** Rog group (HCC patients treated with regorafenib). The Spearman correlation coefficient (r) and p-value are indicated in each panel. A p-value P< 0.05 is considered significant.


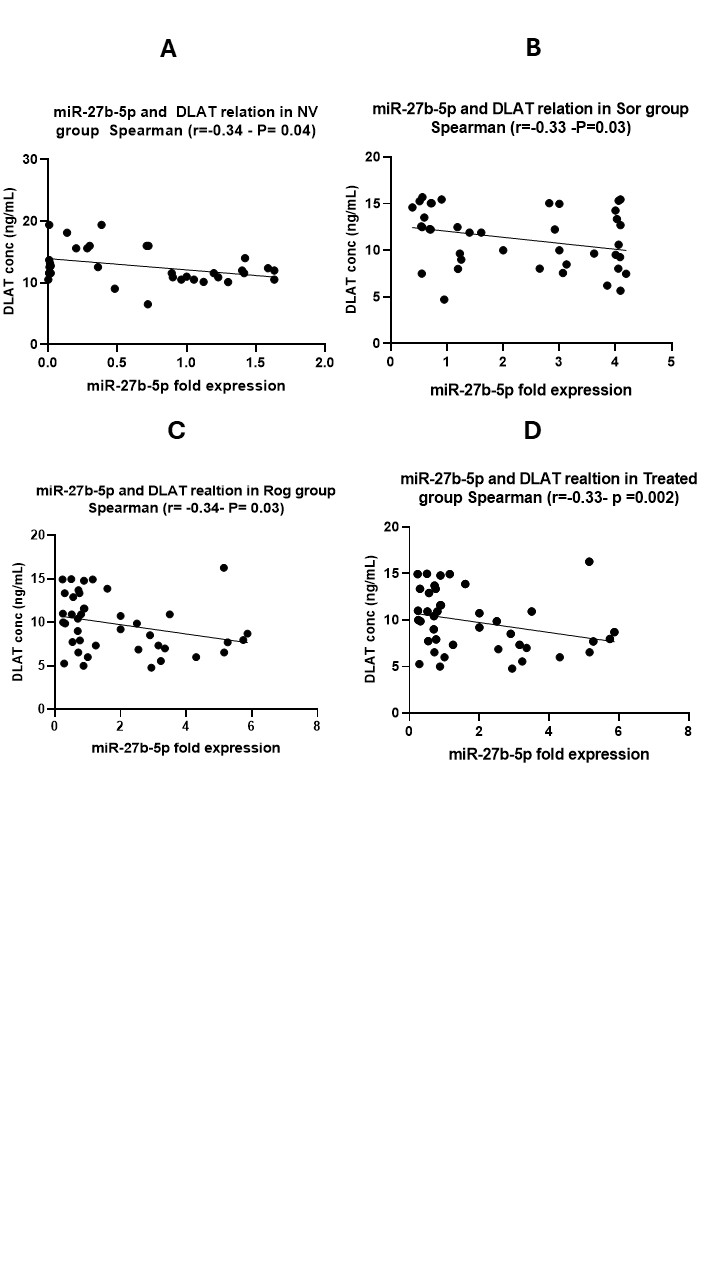


**Fig. S4:** **Correlation analysis Between miR-27b-5p Fold Expression and DLAT Concentration in Different Groups.**

Spearman correlation analysis between miR-27b-5p Fold Expression and DLAT Concentration is presented for the following groups: **A:** NV group (untreated HCC patients), **B:** Sor group (HCC patients treated with sorafenib), **C:** Rog group (HCC patients treated with regorafenib), and **D:** Treated group (HCC patients treated by sorafenib or regorafenib). The Spearman correlation coefficient (r) and p-value are indicated in each panel. A p-value P< 0.05 is considered significant.


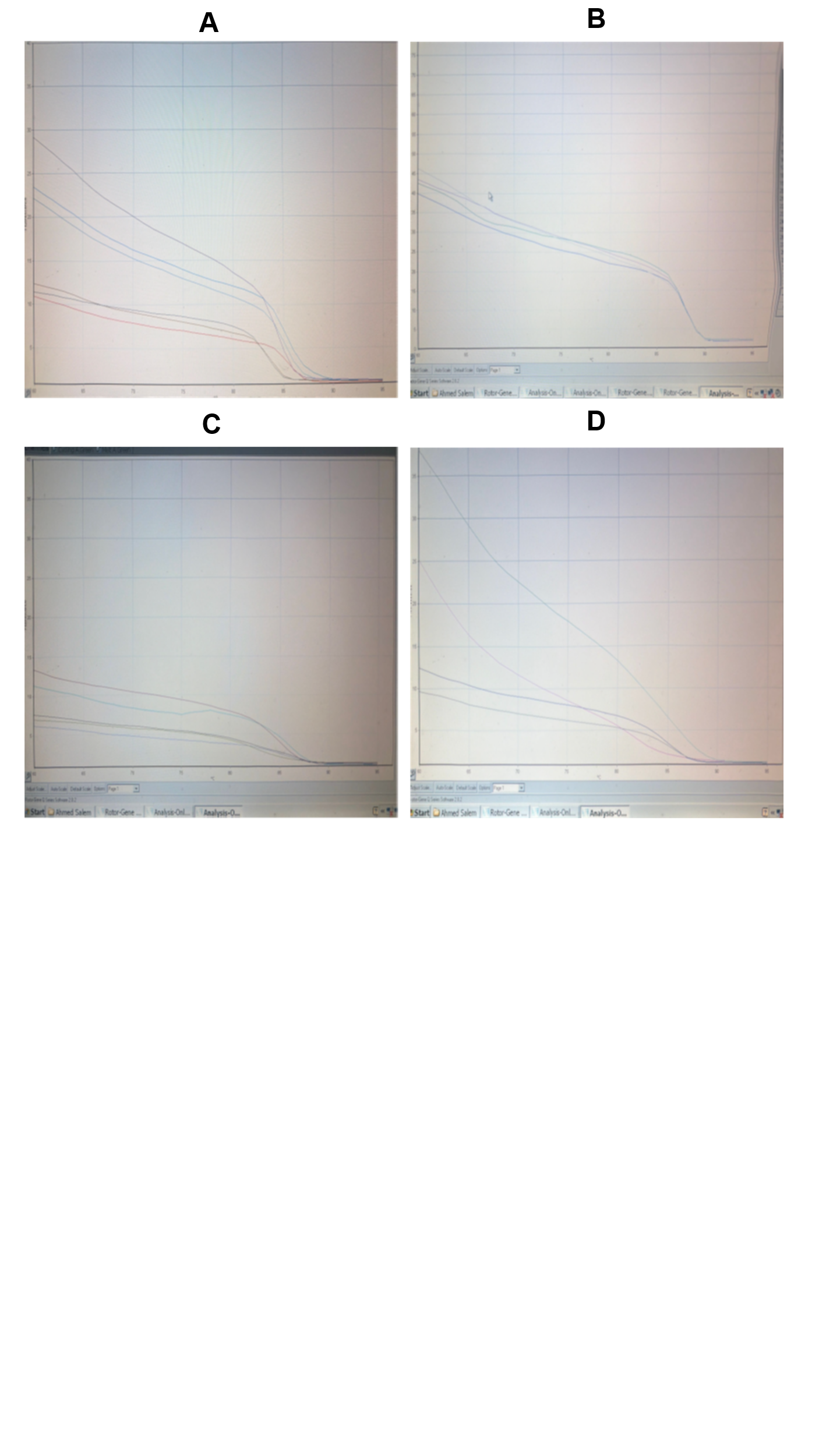


**Fig. S5: Melt curve for studied genes, A: *SLC31A1*, B: GAPDH, C: *ATP7A* & D: *ATP78***

**Table S1: Correlation between KCNQ1OT1/ miR-27b-5p / DLAT axis and some clinical & biochemical parameters in studied groups**

| Biomarker | Group | Correlation Pair | r | p-value |
| --- | --- | --- | --- | --- |
| DLAT | **NV** | Creatinine | –0.4094 | 0.0146 |
| DLAT |  | Metastasis presence | 0.44 | 0.043 |
| KCNQ1OT1 |  | CHILD | 0.3396 | 0.0344 |
| DLAT | **Sor** | AST | –0.3441 | 0.0430 |
| KCNQ1OT1 |  | Albumin | 0.4841 | 0.0091 |
| DLAT | **Rog** | Bilirubin | 0.3570 | 0.0449 |
| DLAT |  | Cause | 0.3811 | 0.0218 |
| KCNQ1OT1 |  | Platelets | –0.4007 | 0.0346 |
| KCNQ1OT1 |  | Bilirubin | 0.5069 | 0.0097 |
| miR-27b-5p |  | AFP | 0.3588 | 0.0343 |
| miR-27b-5p |  | Tumor size | –0.39 | 0.0300 |
| miR-27b-5p |  | Metastasis presence | –0.52 | 0.0030 |

The Spearman correlation coefficient (r) and p-value are indicated in each panel. A p-value p< 0.05 is considered significant.

AST: Aspartate transaminase, AFP: Alpha-fetoprotein, CHILD: Child–Pugh score.
